# Application of Stem Cells in Stroke: A Multifactorial Approach

**DOI:** 10.3389/fnins.2020.00473

**Published:** 2020-06-09

**Authors:** Manisha Singh, Pranav K. Pandey, Ashu Bhasin, M. V. Padma, Sujata Mohanty

**Affiliations:** ^1^Stem Cell Facility (DBT-Centre of Excellence for Stem Cell Research), All India Institute of Medical Sciences, New Delhi, India; ^2^Dr. Solomon H. Snyder Department of Neurosciences, Johns Hopkins University, Baltimore, MD, United States; ^3^Dr. R.P. Centre for Ophthalmic Sciences, All India Institute of Medical Sciences, New Delhi, India; ^4^Department of Neurosciences, All India Institute of Medical Sciences, New Delhi, India

**Keywords:** stroke, stem cells, mesenchymal stem cells, clinical trials, pre-clinical studies

## Abstract

Stroke has a debilitating effect on the human body and a serious negative effect on society, with a global incidence of one in every six people. According to the World Health Organization, 15 million people suffer stroke worldwide each year. Of these, 5 million die and another 5 million are permanently disabled. Motor and cognitive deficits like hemiparesis, paralysis, chronic pain, and psychomotor and behavioral symptoms can persist long term and prevent the patient from fully reintegrating into society, therefore continuing to add to the costly healthcare burden of stroke. Regenerative medicine using stem cells seems to be a panacea for sequelae after stroke. Stem cell-based therapy aids neuro-regeneration and neuroprotection for neurological recovery in patients. However, the use of stem cells as a therapy in stroke patients still needs a lot of research at both basic and translational levels. As well as the mode of action of stem cells in reversing the symptoms not being clear, there are several clinical parameters that need to be addressed before establishing stem cell therapy in stroke, such as the type of stem cells to be administered, the number of stem cells, the timing of dosage, whether dose-boosters are required, the route of administration, etc. There are upcoming prospects of cell-free therapy also by using exosomes derived from stem cells. There are several ongoing pre-clinical studies aiming to answer these questions. Despite still being in the development stage, stem cell therapy holds great potential for neurological rehabilitation in patients suffering from stroke.

## Introduction

Stroke is one of the leading causes of chronic disability and mortality, with 102 million disability-adjusted life years lost annually (Steven, 2008). The Global Burden of Disease, Injuries, and Risk Factors Study (GBD 2015) reported a shift from communicable diseases toward non-communicable diseases like cerebrovascular events. While the incidence of stroke is decreasing in the developed world, it has peaked in low- and middle-income countries like India due to demographic transition and rapid shifts in the socioeconomic milieu (Thomson, 1998). The estimated adjusted prevalence rate of stroke is reported to have a range of 84–262/100,000 in rural and 334–424/100,000 in urban India (Wichterle et al., 2002; Nagai et al., 2010).

The only neuroprotective agent developed for stroke in clinical use is recombinant tissue plasminogen activator (rtPA), which is employed for thrombolysis and has a therapeutic window of merely 3–4.5 h. There is thus a compelling need to develop therapeutic agents that extend beyond the first few hours after onset of stroke. This requires a paradigm shift to the usage of new strategies from neuroprotection to neuro-restoration that treat the injured or compromised brain tissue.

The majority of stroke survivors are left with some degree of disability, particularly upper limb dysfunction, despite several neurorehabilitation therapies. Physical therapy incorporating exercises, motor learning principles, motor cortex stimulation (using rTMS, TDCS), and assistive technologies aid the restoration of functional movements (Tae-Hoon and Yoon-Seok, 2012). The emergence of regenerative medicine has fueled interest across readers and clinicians to study its potential. Over the last decade, an enormous amount of work has been done exploring the potential of a variety of cells like adult stem cells, umbilical cord blood, and cells from adipose tissue and skin.

### Pattern of Stroke Recovery

The recovery after stroke has been explained as a rich cascade of events encompassing cellular, molecular, genetic, demographic, and behavioral components. Such factors have been proven as covariates in therapeutic trials of restorative agents with a sound neurobiological basis. Advances in functional neuroimaging and brain mapping methods have provided a valuable parallel system of data collection for stroke recovery in humans. The recovery in a stroke-affected individual will largely depend on the size of lesion, the internal milieu of the brain injury, and the age and comorbid status of the patient. In general, the first epoch encompasses the initial hours after a stroke, when rapid change occurs in blood flow, edema, pro-inflammatory mechanisms. A second epoch is related to spontaneous behavioral recovery, which begins a few days after stroke onset and lasts several weeks. During this epoch, the brain is galvanized to initiate repair, as endogenous repair-related events reaching peak levels, suggesting a golden period for initiating exogenous restorative therapies. A third epoch begins weeks to months after stroke, when spontaneous behavioral gains have generally reached a plateau, and this stable state is responsive to many restorative interventions (Steven, 2008).

### Mechanisms of Action of Stem Cells in Neural Repair

**FIGURE 1:**
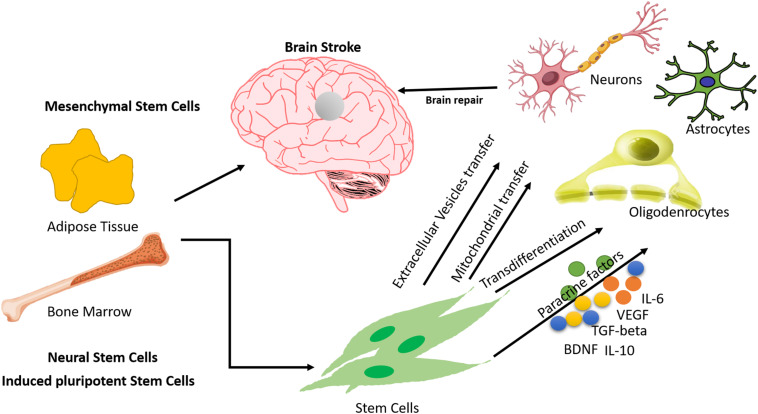
Mechanisms of action of Mesenchymal Stem Cells in treating stroke.

Stem cells have the capacity to differentiate into all types of cells. Exogenously administered cells appear to stimulate endogenous reparative processes and do not replace injured cerebral tissue. It was once thought that intravenously administered cells would home in on the injured site and replace the dead neurons, but the current ideology for the use of these cells holds that these cells release many trophic factors like VEGF, IGF, BDNF, and tissue growth factors that stimulate brain plasticity and recovery mechanisms. Upregulation of growth factors, prevention of ongoing cell death, and enhancement of synaptic connectivity between the host and graft are some of the common pathways through which intravenous stem cells work as “chaperones.” Regarding the timing of transplantation, preclinical studies have shown that cell therapy increases functional recovery after acute, sub-acute, and chronic stroke (Bliss et al., 2010), but few studies have compared different time windows, with differing results according to the model system and cell type studied. All of the possible modes of action of stem cells have been described in Figure 1.

## Translational Approach for the Development of Regenerative Medicine in Brain Stroke

The unique capacity of stem cells of self-renewal and differentiation has been exploited to devise cell-based therapy for various neurodegenerative diseases, including brain stroke. There have been several studies, which will be discussed in the upcoming paragraphs, that report the use of stem cells in the treatment of various diseases. These studies have used stem cells of various kinds, such as adult stem cells (mesenchymal stem cells and neural stem cells), embryonic stem cells, and the latest kind, induced pluripotent stem cells. Apart from using different types of stem cells, scientists have also reported distinctive modes of action to support their study outcomes. Besides these variable points, there are other considerations like the dosage of stem cells, mode of administration of stem cells, and whether or not booster doses are required, depending upon the magnitude of the disease. Various groups have attempted to answer these vital questions through their research.

Ischemic stroke causes severe damage to the brain cells by destroying the heterogeneous cell population and neuronal connections along with vascular systems. The regenerative potential of several types of stem cells like embryonic stem cells, neural stem cells, adult stem cells (Mesenchymal stem cells), and induced pluripotent stem cells have been assessed for treating stroke. The outcomes and observations in these studies are not consistent. Most of the studies have only commented on the homing, survival, proliferation, and differentiation of stem cells on the site and their limited neuro-restorative ability. Embryonic stem cells (ESCs) are pluripotent cells derived from the inner cell mass of the blastocyst. There have been a few studies where engraftment of murine ESCs in mouse models of ischemia has led to the restoration of behavioral deficits, synaptic connections, and damaged neurons (Thomson, 1998; Wichterle et al., 2002; Nagai et al., 2010). However, the use of ESCs in the clinical setting is argued against by many other groups due to their immunogenic nature and teratoma-forming tendency (Fong et al., 2010; Kawai et al., 2010; Ghosh et al., 2011). Hence, scientists are now trying to establish the neuro-restorative ability of other stem cell types. Neural stem cells (NSCs) are theoretically the most appropriate cell candidates for neuro-restoration as they belong to the same tissue source and have a natural tendency to differentiate into neuronal cells. NSCs are multipotent cells that are generally found in the subgranular zone of the dentate gyrus of the hippocampus (Toda et al., 2001). Engraftment of NSCs has been reported to lead to the reformation of synaptic connections and improvement in the electrophysiological properties of mature neurons in the damaged brain (Polezhaev and Alexandrova, 1984; Polezhaev et al., 1985; Cho et al., 2002; Oki et al., 2012). They do so by improving the extracellular microenvironment and hence encouraging neuronal circuit plasticity (Ourednik et al., 2002; Lee et al., 2007; Redmond et al., 2007; Jeyakumar et al., 2009). NSCs restore neuronal functions as they secrete several neurotrophic factors like BDNF and VEGF, which help in maintaining the health, generation, proliferation, and survival of the neurons, along with the maintenance of ECM (Emanueli et al., 2003; Jung et al., 2008; Lee H. J. et al., 2010; Smith et al., 2012). VEGF specifically helps in angiogenesis and vascular restoration of the blood vessels damaged due to ischemia (Song et al., 2015; Ryu et al., 2016). CNTF, GDNF, NGF, and other such factors secreted by NSCs also play vital roles in the protection, maintenance, and proliferation of neural cells (Abe, 2000).

Another type of cells with amazing neuro-restorative potential and that have several other desirable properties, like being immunologically naive, easy to extract and maintain and expand *in vitro*, and not having associated ethical concerns, are mesenchymal stem cells (MSCs) (Baksh et al., 2007; Uccelli et al., 2008; Russell et al., 2018). MSCs are multipotent stem cells that have their niche in body tissues like bone marrow, adipose tissue, umbilical cord, umbilical cord blood, dental pulp, etc (Uccelli et al., 2008; Singh et al., 2017; Russell et al., 2018). Extracting MSCs from these tissues is a very well-established and easy process and has been very widely used in various clinical trials (Nandy et al., 2014; Singh et al., 2017). MSCs lead to neuro-restoration by one or more modes of action such as the release of paracrine factors, cell replacement, mitochondrial transfer, etc. MSCs also have an angiogenic effect. They have been reported to induce angiogenesis by the release of vascular endothelial growth factor (VEGF) (Li et al., 2000, 2001; Chen et al., 2003; Shen et al., 2007). The only issue to be considered for using bone marrow-derived MSCs is the surgical intervention to obtain the bone marrow. Adipose tissue-derived MSCs have proved to be equally effective in neuro-regeneration, with the added advantages of being easily accessible and more abundant (Yang et al., 2012; Moore and Abrahamse, 2014; Singh et al., 2017). Adipose tissue-derived MSCs have been known to play a protective role through the release of extracellular vesicles. There are studies reporting the safety and efficacy of extracellular vesicles derived from adipose tissue-derived MSCs (Ra et al., 2011; Zhang Y. et al., 2015; Chen et al., 2016; Bang and Kim, 2019). However, more detailed studies are required to establish MSCs as therapeutic agents.

Another type of stem cell that has been explored for its translational value recently is the induced pluripotent stem cell (iPSC). There has been a boom in research into iPSCs after the groundbreaking discovery by Takahashi and Yamanaka (2006). iPSCs have the edge over other types of stem cells due to being non-immunogenic, easy to access, and non-interventional and not giving rise to ethical concerns. However, their generation is still an unresolved issue, as the reprogramming efficiency is still very low. Additionally, some studies have reported the formation of teratoma in the mouse brain, which implies that the tumorigenicity of iPSCs needs to be addressed and resolved before taking them into the clinical setting. iPSCs seem to be formidable stem cells for tissue regeneration (Israel et al., 2012; Fernández-Susavila et al., 2019).

### Bioactive Constituents in Brain Stroke: Combination Therapy

The use of complementary and alternative medicine along with stem cell therapy also plays an important role in the recovery of brain stroke patients. During the stroke episode, most of the pro-inflammatory cytokines are involved, and many polyphenol compounds extracted from different parts of medicinal plants have been shown to protect against cerebral ischemia in pre-clinical models. Glycrrhizin extracted from the licorice root, *Glycrrhiza glabra*, protected against the rat brain injury induced by stroke. Intraperitoneal administration of Glycrrhizin pre- and post-stroke helped inhibit the infarction by ameliorating the IFN-γ mediated T-cell activity, which was partially modulated by high mobility group box-1 (Xiong et al., 2016). The use of intravenous administration of recombinant plasminogen tissue activator (rtPA) was approved half a decade ago, but the limitations to rtPA treatment include a narrow therapeutic window of 4.5 h post-stroke and a high risk for hemorrhagic transformations. MSC transplantation in brain stroke patients is an existing approach, but inflammation has sometimes been observed in MSCs due to oxygen glucose deprivation during treatment. One study showed that a nano-formulation of gelatin-coated polycaprolactone loaded with naringenin, a strong anti-inflammatory, protected the MSCs against oxygen glucose deprivation-induced inflammation and also reduced the levels of pro-inflammatory cytokines (TNF-α, IFN-γ, and IL-β) and of the anti-inflammatory biomarkers COX-2, iNOS, and MPO (Ahmad et al., 2019). The active compound Eugenol, isolated from *Acorus gramineus*, was tested in a cerebral ischemia perfusion rat model. Pre-treatment with Eugenol in the rat model showed that it was prompt in attenuating cerebral ischemic injury by inducing autophagy via the AMPK/mTOR/P70S6K signaling pathway. In another study, the neuroprotective effect of quercetin was demonstrated in mice, and the findings suggested that the quercetin helped reduce apoptosis in the focal cerebral ischemia rat brain and that the mechanism may be related to the activation of the PI3K/Akt signaling pathway (Yao et al., 2012). The intragastric administration of berberin and glycyrrhizin showed neuroprotective effects in mice subjected to transient middle cerebral artery occlusion. The co-administration of glycyrrhizin and berberin showed more potent suppression on the HMGB1/TLR4/NF-kB pathway in comparison to treatment with either alone. The results of the study suggested that the administration of these compounds protects the brain from ischemia-reperfusion injury and that the mechanism may rely on their anti-inflammatory effects and, moreover, also by suppressing the activation of the HMGB1/TLR4/NF-kB signaling pathway (Zhu et al., 2018). Medicinal plants contain several important bioactive constituents that help in several modalities. Numerous pre-clinical studies have been performed using plant-derived products that help modulate the proliferation and differentiation of MSCs, as well as being useful in the field of biomaterials. Therefore, the new combination therapy of phytochemicals along with stem cell therapy may become a new perspective in stem cell-based neuro-regeneration.

## Pre-Clinical Studies With Stem Cells in Brain Stroke

The experimental evidence of the benefits of stem cells in treating stroke has been provided over the course of several years (Abe, 2000; Mays et al., 2010). The usefulness of various types of stem cells has been proclaimed in various neurological diseases, along with their safety and efficacy at both pre-clinical and clinical levels. The pre-clinical validation of stem cells in treating stroke has been instrumental. Various study groups have validated the use of stem cells in terms of various parameters such as type of stem cells, number/dose of stem cells, mode of administration, homing and tracking of stem cells, and safety and efficacy of stem cells (Zheng et al., 2018; Borlongan, 2019).

The most commonly used and most widely explored stem cells in the treatment of stroke are MSCs. Among the various tissue sources of MSCs, the most common and widely explored are bone marrow and adipose tissue, with bone marrow being the oldest of all (Andrews et al., 2008; Xin et al., 2013; Zhang et al., 2014; Zhang Y. et al., 2015). However, neural stem cells and bone marrow-derived mononuclear stem cells have also been explored (Taguchi et al., 2004; Darsalia et al., 2007; Takahashi et al., 2008). In most of the pre-clinical studies, autologous bone marrow-derived MSCs have been used (Zhang et al., 2006; Khalili et al., 2012; Otero et al., 2012; Bao et al., 2013; Vaquero et al., 2013) to investigate the various aspects of stem cell transplantation in stroke. Several other studies report the use of MSCs from other tissue sources, like adipose tissue, umbilical cord, placenta, etc (Yang et al., 2012; Zhang Q. et al., 2015; Xie et al., 2016). MSCs are characterized for transplantation based on surface marker profiling, which includes the presence of markers like CD29, CD44, CD73, CD90, and CD105 and the absence of CD34/45, CD14, and HLA class II. Other critical factors that need to be considered for pre-clinical studies are the number/dose of cells to be administered and the mode of administration. Transplantations of MSCs range from 1 × 10^6^ to 8 × 10^6^ cells and are accomplished through different modes, including intravenous, intranasal, and intra-arterial (Chen et al., 2001; Shyu et al., 2006; Zhang et al., 2006; Yang et al., 2012; Ma et al., 2016; Rodríguez-Frutos et al., 2016; Borlongan, 2019). While there is evidence that the transplanted MSCs have homed and differentiated into neurons, astrocytes, and oligodendrocytes upon administration through intravenous, intranasal, and intracerebral modes, there are doubts on the migration of MSCs in the brain by the intravenous mode (Díez-Tejedor et al., 2014). Also, there are mixed reports on whether the transplantation of coaxed and naive stem cells can achieve the desired outcome in terms of functional recovery, BBB function, increased angiogenesis and vasculogenesis, and tissue regeneration (Laso-García et al., 2019; Turnbull et al., 2019). More detailed studies need to be done to establish a definitive stem cell therapy regime for stroke.

## Clinical Trials of Regenerative Medicine in Brain Stroke

Cerebrovascular strokes can cause morbidity and mortality and induce long-term disability that affects quality of life. Stroke is associated with neuroinflammation, which plays a key role in the pathophysiology of cerebrovascular accidents of different types. We performed a rigorous search of a database on clinical studies with stroke and found more than 56 clinical trials on the use of regenerative medicine (autologous or allogeneic) for cerebrovascular stroke. Most of them used mesenchymal stem cells, adipose tissue, bone marrow-derived cells, and spinal cord and umbilical cord cells. Table 1 presents a few clinical trials involving stem cell therapy (autologous and allogeneic), giving their study design, dose, route of administration, and outcomes. Our experience with regenerative medicine in stroke emphasizes the safety and tolerance of cells, whereas efficacy still needs to be addressed. More recovery in clinical and functional patterns was observed in patients administered with autologous bone marrow-derived cells than in the group with physiotherapy alone. We also tried to elucidate correlations between functional MRI and outcome after stroke, with increased activation in premotor and primary motor areas (PM and SMA), and contralesional M1 over activation. Our present randomized controlled trial studying the paracrine effects of autologous mononuclear stem cells in interim showed increased VEGF and BDNF post-treatment in all stroke patients, suggesting endogenous recovery after restorative therapies like stem cells and a structured neuro-rehabilitation regime. To counter the progression of the cerebral vascular disease post-stroke and repair the damage induced in different regions of the brain, various clinical trials with different stem cells like mesenchymal stem cells, adipose tissue-derived stem cells, and bone marrow mononuclear stem cells are ongoing (Table 1) that investigate potential efficacy and safety, without the occurrence of any adverse or severely adverse events.

**TABLE 1:** List of Clinical trials using Stem cells in treating stroke.

**Country and year**	**Study design**	**Sample size**	**Stroke type**	**Cells from**	**Interventional type**	**Route of administration**	**Dose**	**Time point from onset of infusion**	**Followup (Months)**	**Primary outcome indicator**	**References**
South Korea, 2010	Open-label, observer-blinded clinical trial	16/36	Acute	Posterior iliac crest	MSCs, Conventional Treatment	Intravenous	50 × 10^6^	7 days	60	mRS	Lee J. S. et al., 2010
Spain, 2012	Single-blind controlled clinical trial phase I/II	10/10	Subacute	Posterior superior iliac crest	BM-MNCs, Conventional Treatment	Intraarterial	159 × 10^6^	Mean 5–9 days post occurrence of stroke	6	NIHSS, BI, mRS	Moniche et al., 2012
India, 2012	Non-Randomized controlled clinical trial	12/12	Chronic	Posterior superior iliac crest	BM-MNCs, Conventional Treatment	Intravenous	50–60 × 10^6^	3 months–2 years post-stroke	6	FMA, BI	Bhasin et al., 2012
India, 2013		20/20	Chronic	Post superior iliac crest	MNC + MSCs	Intravenous	50–60 × 10^6^	3 months–2 years post-stroke	6	BI, FMA	Bhasin et al., 2016
China, 2013		60/60	Chronic	Umbilical cord	UC-MSCs	Subarachnoid + Intravenous	100 × 10^6^	1 month and 6 years post-stroke	3	FMA	Hu et al., 2013
India, 2014	Multicentric, phase II parallel group controlled clinical trial	60/60	Subacute	Posterior iliac crest	BMSCs	Intravenous infusion	280.5 × 10^6^	7–30 days	6	NIHSS, BI	Prasad et al., 2014
China, 2014	Randomized, single-blind clinical trial	15/15	Chronic	PBSCs	PBSCs	Stereotactic	3–8 × 10^6^	6 month–5 years	12	NIHSS, mRS	Chen et al., 2014
China, 2014		50/50	Subacute	Lianoning blood centers	UC-MSCs, Conventional treatment	Subarachnoid	100 × 10^6^	14–30 days post-stroke	3	NIHSS,FMA	Feng et al., 2014
Spain, 2014	Phase IIa, Prospective, randomized, double-blind, placebo-controlled, single-center, pilot clinical trial.	20/20	Acute	Adipose tissue	Allogenic MSCs from adipose tissue	Intravenous administration	1 million/unit/kg. body weight	2 weeks post-stroke	24	mRS, NIHSS, MRI, Biochemical markers	Díez-Tejedor et al., 2014
USA, 2018	Phase I, Open label	10/10	Acute, cortical ischemic stroke	Allogenic Umbilical cord	Umbilical cord blood infusion	Intravenous administration	3.34 × 10^6^	3–9 days	12	mRS	Laskowitz et al., 2018
USA, 2019	Phase I, Single arm-	25/30	Acute	Bone marrow harvest (autologous)	B-MNCs	Intravenous administration	10 million cells/kg b.wt	1–3 days	24	mRS, NIHSS	Vahidy et al., 2019
China, 2019	Cohort (Phase I)	9	Hemiparesis from ischemic stroke	Cells derived from human fetal spinal cord	NSI-566, primary adherent neural stem cell	Intracerebral injection	Cohort A = 1.2 × 10^7^ Cohort B = 2.4 × 10^7^ Cohort C = 7.2 × 10^7^	Mean 494 days	24	CT, fMRI, PET, DTI	Zhang et al., 2019
California, 2019	Phase I/II Preliminary safety and efficacy studies	Phase I (*n* = 15) Phase II (*n* = 21)	Chronic		Allogeneic Ischemia tolerant MSCs	Intravenous transfusion	Phase I (0.5, 1.0, and 1.5 million/kg b.wt) Phase II 1.5 million/kg b.wt		12	BI, ECG, CT-Scan.	Levy et al., 2019

**mRS, Modified Rankin Score; MRI, Magnetic resonance imaging; PET, Positron emission tomography; CT, Computed tomography; NIHSS, National Institute of Health Stroke scale; BI, Barthel Index.**

An open-labeled observer-blind clinical trial was conducted to evaluate the long-term safety and efficacy of autologous MSCs. Post-transplantation with MSCs, clinical improvement in patients was observed in the MSC-treated patient group, which was associated with the serum level of stromal cell-derived factor-1 and the degree of involvement of the sub-ventricular region of the lateral ventricle. No serious adverse effects were observed during long-term follow up of patients. The occurrence of comorbidities was similar in comparison to the control group (Lee J. S. et al., 2010). In another single-blind controlled phase I/II trial, patients with middle cerebral artery stroke were enrolled in the study. Autologous bone marrow mononuclear cells (BM-MNCs) were injected 5–9 days post-stroke. A higher plasma β-nerve growth factor level was observed post-injection, and no adverse events were observed for 6 months apart from two patients in whom partial seizures were observed at 3 months of follow up. The study result suggested that intra-arterial administration of BM-MNCs is safe and feasible (Moniche et al., 2012). A non-randomized observational controlled study with autologous bone marrow mononuclear cells in chronic ischemic stroke showed better efficacy and did not observe any adverse effects or neurological abnormalities during long-term follow up of patients (Bhasin et al., 2012). Intravenous administration of autologous BM-MSCs was also shown to have better safety in a randomized, phase II, multicentric trial group in patients with subacute ischemic stroke (Prasad et al., 2014). On the basis of the findings of pre-clinical studies with peripheral blood stem cells (PBSCs), randomized single-blind controlled studies were conducted in patients with middle cerebral artery infarction. Patients were enrolled as per the inclusion criteria of the study and received subcutaneous G-CSF injection for 5 consecutive days prior to stereotaxic implantation of immune-sorted PBSCs. No adverse events were observed during the study procedure or the follow up of the study. Clinical outcomes of the PBSC-treated and control groups were observed in terms of changes in NIHSS, ESS, EMS, and mRS from baseline to 12 months. Moreover, this study also provided important evidence on the efficacy of PBSCs in improving stroke-related motor deficits, the reconstruction of injured CST, and the rebuilding of electrophysiology activity from the brain to the limbs (Chen et al., 2014). Intravenous administration of allogeneic mesenchymal stem cells from adipose tissue in a phase II randomized, double-blind, placebo controlled single-center pilot clinical trial in patients 2 weeks post-acute stroke showed better efficacy without the occurrence of adverse events. Moreover, the use of allogenic MSCs could be an alternative therapy for the treatment of stroke because it has been demonstrated that they lack class II HLA antigens (Díez-Tejedor et al., 2014). Another study (Bhasin et al., 2016) reported the paracrine mechanism of bone marrow-derived mononuclear cells in chronic ichemic stroke patients. CD34+ was counted in BM-MNCs for each and every patient. Intravenously administered BM-MNCs secrete glial cell-derived neurotrophic factor and BDNF, IGF-1, and VGEF, which may protect against the dysfunction of motor neurons. The trial results suggested that the administration of BM-MNCs is safe and feasible for stroke patients. In another phase I, open-label, prospective clinical trial, patients with acute ischemic stroke received a single i.v. infusion of allogeneic human umbilical cord blood cells within a window of 3–10 days. Post-UCB infusion, graft-vs.-host disease, infection, and hypersensitivity were analyzed at patient follow up visits at 3, 6, and 12 months. Adverse events and severe adverse events (AE/SAE) in the patients that were directly or indirectly related to the investigational treatment were reported (Laskowitz et al., 2018).

A single-arm, phase I clinical trial study of autologous bone marrow mononuclear cells for acute ischemic stroke showed a promising new investigational modality that may help widen the therapeutic window for patients with ischemic stroke. AEs/SAEs were observed post-transplantation, some of which may have been associated with the intervention but others of which may not have (Vahidy et al., 2019). In another single-site phase I study, the feasibility and safety of NSI-566 primary adherent neural cells derived from a single human fetal spinal cord were observed. Three different doses were investigated in a cohort study of patients, and it was shown that the transplantation of human spinal cord-derived neural stem cells into the peri-infarct area of stable stroke patients is beneficial. The mechanism potentially behind it is that the stem cell-derived tissue is largely composed of interneurons and glial cells, and these promote regeneration and act as bridges between regenerating neuronal fibers (Zhang et al., 2019). A phase I/II preliminary safety and efficacy study of allogenic MSCs in chronic stroke patients showed the dose tolerability to be 1.5 million/kg body weight in phase I and phase II study. The primary outcome of intravenous administration of allogenic MSCs in patients was measured for 1 year, and secondary outcomes were measured in terms of behavioral changes. AEs/SAEs were observed in 13 patients that were probably not related to the intervention, and two mild AEs related to the study intervention were observed, urinary tract infection and intravenous site irritation. However, other mechanisms have also been shown that involve cell replacement, immunomodulatory action, and endogenous repair of brain damage post-stroke. The stem cell therapy in cerebrovascular accident depends overall upon their differentiation, inflammation, and ability to repair of endogenous processes. This regenerative medicine has emerged as an important tool in modern neurology, with potential efficacy in neurodegenerative disorder (Thwaites et al., 2012; Yu et al., 2013). After extensive findings of pre-clinical research, the clinical trials have conducted with different stem cells in stroke, in which the AEs/SAEs observed during or post transplantation may be directly or indirectly related to the intervention. The studies suggest that there must be a further continuation of pre-clinical and clinical studies of regenerative medicine in stroke patients to further elucidate the safety, efficacy, and toxicity pre and posting transplantation and their capacity to deliver potent efficacious regenerative medicine for stroke patients. Further clinical trials of regenerative medicine in cerebrovascular stroke are complete, with more results awaited.

### Future Prospects

Regenerative medicine is looking increasingly more enticing as we capture more evidence from past and current clinical trials in stroke (Bhasin et al., 2016, 2017). The neurophysiology describing stem cells and their concatenated mechanisms suggests that restoration of brain function may be a realistic goal. There are several cellular labeling techniques available, including simple incubation, use of transfection agents, magnetoelectroporation, and magnetosonoporation. MR tracking with SPIOs and nanoparticles in a MCAo occlusion model of stroke has proven flawless in tracking cells but still needs clinical validation (Cromer Berman et al., 2011). To make this research a therapeutic boon in stroke, certain questions still need answers, such as the optimal cell delivery route, the initial engraftment and distribution pattern of injected cells, and how effectively injected cells migrate toward the affected sites.

While stem cells have proven to be a great resource for treating stroke, there are still several obstacles to be conquered in the near future. A variety of stem cells with multiple parameters have been under trial for the treatment of stroke. Starting from the kinds of stem cells in use, there are pluripotent stem cells (ESCs and iPSCs), neural stem cells, and adult stem cells (MSCs from various tissues). There are ethical concerns associated with pluripotent stem cells. Additionally, NSCs have limitations in their *in vitro* expansion (in terms of the number of NSCs required to be transplanted). MSCs are capable of combating this concern. Another issue is immunological tolerance between the host body and transplanted stem cells. This issue can be resolved by using the patient’s own cells to derive iPSCs of MSCs (as they are devoid of HLA class II). Besides these concerns, there are several other concerns, such as whether the efficiency of cell extraction, expansion, and differentiation is sufficient for transplantation, as well as the best mode of injection and optimal number of injections. While there are several challenges to bringing stem cell therapy in the mainstream of treatment for various diseases, stem cell therapy has been established for treating several degenerative and other kinds of diseases. In future, all these points of concern need to be addressed to make stem cell therapy an abiding treatment regime for stroke.

## Author Contributions

MS, AB, and PP: drafting and refining the manuscript. SM, MS, and AB: critical reading of the manuscript. All of the authors have read and approved the manuscript.

## Conflict of Interest

The authors declare that the research was conducted in the absence of any commercial or financial relationships that could be construed as a potential conflict of interest.
